# Economies through Application of Nonmedical Primary-Preventative Health: Lessons from the Healthy Country Healthy People Experience of Australia’s Aboriginal People

**DOI:** 10.3390/ijerph13040400

**Published:** 2016-04-01

**Authors:** David Campbell

**Affiliations:** Centre for Remote Health, Flinders University, P.O. Box 4066, Alice Springs, NT 0871, Australia; d.campbell@flinders.edu.au; Tel.: +61-8-8951-4744; Fax: +61-9-8951-4777

**Keywords:** chronic disease pandemic, Indigenous, social benefit, psychosocial stressors, environmental benefit, noncommunicable disease

## Abstract

The World Health Organization reports noncommunicable disease as a global pandemic. While national and international health research/policy bodies, such as the World Health Organization and the Australian Institute of Health and Welfare, emphasize the importance of preventative health, there is a continuing distortion in the allocation of resources to curative health as a result of government failure. Government failure is, in part, the result of a political response to individual preference for certainty in receiving treatment for specific health conditions, rather than the uncertainty of population-based preventative intervention. This has led to a failure to engage with those primary causative factors affecting chronic disease, namely the psychosocial stressors, in which the socioeconomic determinants are an important component. Such causal factors are open to manipulation through government policies and joint government-government, government-private cooperation through application of nonmedical primary-preventative health policies. The health benefits of Aboriginal people in traditional land management, or caring-for-country, in remote to very remote Australia, is used to exemplify the social benefits of nonmedical primary-preventative health intervention. Such practices form part of the “healthy country, health people” concept that is traditionally relied upon by Indigenous peoples. Possible health and wider private good and public good social benefits are shown to occur across multiple disciplines and jurisdictions with the possibility of substantial economies. General principles in the application of nonmedical primary-preventative health activities are developed through consideration of the experience of Afboriginal people participation in traditional caring-for-country.

## 1. Introduction

This paper concerns the imbalance between curative and nonmedical primary-preventative health. While substantial health gains are attributed to nonmedical primary-preventative health [1,2,3,4], there is an apparent lack of systematic coordination of such aspects in the overall management of health outcomes. The application of nonmedical primary-preventative health is shown to be effective in the prevention and mitigation of noncommunicable or chronic disease among Australia’s Aboriginal people. This experience is relevant to correcting the health disadvantages suffered by Australia’s Aboriginal and Torres Strait Islander peoples and the world population of Indigenous people. At a higher level of abstraction, nonmedical primary-preventative health is relevant to the prevention and mitigation of the global chronic disease pandemic [3,4,5,6].

Given the global noncommunicable disease pandemic [5,7], the application of preventative health is particularly important at this time. National and international health research/policy bodies, such as the World Health Organization [5,6,7] and the Australian Institute of Health and Welfare [8,9], acknowledge the importance of preventative actions, especially when it comes to the socioeconomic determinants of health. In spite of the strong argument made for a commensurate response to the socioeconomic determinants, there has “… been a failure for a commensurate response” [10] (p. 138). Bryant *et al.* [11], in the case of Canada, and Fisher *et al.* [12], in the case of Australia, commented on this failure, and noted an apparent preference by policy makers to focus on the delivery of health care/curative health. As explained in the following, economists, such as Keech and Munger [13], and Watts and Segal [14], identify such resource misallocation as government policy failure.

Use of the term “primary” is in reference to the primary causative agents, or stressors affecting the increasing incidence of noncommunicable disease [15,16,17,18,19,20,21]. Early and ongoing nonmedical life factors affect noncommunicable health outcomes. These factors include a range of social, economic (the socioeconomic determinants) and psychological factors including lack of access to and control over resources, social isolation, social disenfranchisement, racism, class distinction, and lack of mastery and control over life circumstances [1,21,22,23,24]. These factors extend beyond the socioeconomic determinants of health and are referred to here as psychosocial stressors, in which the causal pathway to noncommunicable disease is emphasized. Depending on individual coping capacity and response, stressors can lead to chronic stress, disruption of the endocrine system and an increase in long term morbidity and mortality risk [25,26]. Cass *et al.* [27], for example, highlighted the role of distal upstream psychosocial stressors as primary causal agents leading to noncommunicable disease, with risky behavioral responses being a secondary causative agent.

On average, less than 3% of the national annual health budgets of the Organization for Economic Cooperation and Development members, including Australia, are allocated to preventative health—with little of this funding allocated to the mitigation of upstream psychosocial stressors [28,29]. Such accounting makes no explicit allowance for expenditures outside of the health budget that might affect health outcomes. These expenditures include housing, education, direct investment in Aboriginal involvement in traditional land management, public transport, access to resources, and services for those who are financially disadvantaged.

### The Experience of Aboriginal People as a Case Study

The burden of chronic disease is unevenly distributed, with the world’s Indigenous peoples suffering a heavier burden of noncommunicable disease [30] (Table 1). This maldistribution includes Australia’s Aboriginal and Torres Strait Islander peoples, who suffer a heavier burden of chronic disease to that suffered by other Australians (Table 1). One approach in addressing this burden among Indigenous peoples is through their engagement in “healthy country, healthy people”. The benefit of this approach is shown on the basis of the results of the involvement of Australia’s Aboriginal people in traditional land management, or caring-for-country1 [31,32,33,34,35,36,37,38].

Such activities are an example of nonmedical primary preventative health. Economic analysis of such activities shows how such actions can be a cost effective approach to the prevention and mitigation of noncommunicable disease among Australia’s Aboriginal people [38]. In addition, at a higher level of abstraction, nonmedical primary-preventative health interventions can be applied as a cost effective means to the prevention and mitigation of the global chronic disease pandemic [3,5,6,7].

The following shows that nonmedical primary-preventative health can reduce the demand and associated costs for health services, including reductions in the health risks of wrongly prescribed pharmaceutical and clinical interventions. People can be expected to live longer and healthier lives, with morbidity being compressed till close to death—thus leading to increased human capital, improved social wellbeing, reduced private and public health costs [39,40,41], increased mean per-capita disposable income, and an expansion of the tax base. This can be achieved while providing a range of nonmedical public good social benefits at no additional cost.

## 2. Context of Aboriginal People Being on Country

The successful application and cost effectiveness of a nonmedical primary-preventative health program will need to meet the contextual and cultural circumstance of the people involved. While the circumstances of Aboriginal people are discussed in this section according to residence in remote to very remote Australia, these contextual circumstances are also relevant to Aboriginal people beyond this region. Aboriginal and Torres Strait Islander people are 3% of the Australian population, or 721,000 people. Aboriginal people suffer higher levels of chronic diseases and a shorter life span than do other Australians [42] (Table 1).

Indigenous Australians have inalienable or exclusive possession of approximately 20% of Australia [43], and 25.4% when non-exclusive rights are included [43]. Nearly all of the area under some form of Aboriginal possession is located in the 70% of Australia that is remote to very remote, with 97% of this area being desert [44]. Aboriginal people constitute a twelfth and a fifth of the semi-arid and arid populations.

Most Aboriginal residents are distributed throughout remote to very remote Australia in family groups or in small settlements, with most non-Aboriginal residents concentrated on mining sites and in the five major population centers [44]. Aboriginal people are accordingly well placed to carry out land management across a large region of Australia. This is especially the case as much of the remote Australian ecological system is an anthropomorphic creation over tens of thousands of years of Aboriginal residency [45,46,47,48,49,50,51]. Since early this century, there has been a shift in social land use expectations from one based on agricultural production to one that includes tourism, conservation and Aboriginal occupancy. These changes in social preference emphasize the continued land management role of Aboriginal people and meet with tourist expectations of cultural contact with traditional occupants [51,52,53].

Noncommunicable disease undermines the capacity of Aboriginal people to fulfill this land management role and their socioeconomic advancement [51]. Psychosocial stressors and natural environmental stressors, including climate change [54], further erode health and capacity to perform land management roles and the delivery of private and public good social benefits. This is especially important in the Northern Territory, where Aboriginal people are approximately 30% of the total population.

Davies *et al.* [55] reviewed the cultural, health, self-determination, close cultural connection with country and the cultural, family and stewardship responsibilities that are met through land management. Non-Aboriginal invasion and settlement in Aboriginal country disrupted these relationships, while contributing to the poor health of Aboriginal people and environmental degradation [56,57]. Following a history of often violent disconnection from country, cultural loss, and perceived and actual loss of control [58], reestablishment of Aboriginal jurisdiction over country, and the uptake and application of traditional cultural practices is disjointed.

Variation in government policies and institutions have a greater impact on personal control and self-mastery than exists for non-Aboriginal peoples [58]. The Commonwealth Government’s ongoing 2007 Northern Territory Emergency Response is an example of this [59]. Government emphasis on curative health and the Prime Minister’s deprecating comment in 2015 that attachment to country is a “lifestyle choice” [60], indicates a misunderstanding of or an ignoring of the possible cultural importance of country to personal wellbeing and health. It also ignores the broader national social benefits that can be achievable through caring-for-country.

## 3. Psychosocial Realities: Causal Factors Affecting Risky Behavioral Choice

“Life exists through maintenance of a complex dynamic equilibrium, termed homeostasis, that is constantly challenged by intrinsic or extrinsic, real or perceived, adverse forces, the stressors” [61] (p. 259). Extended stress as a result of the psychosocial stressors and corresponding disruption of the homeostasis system leads to a range of noncommunicable disease and cognitive disorders [16,18,62].

Stressors result from multiple endogenous and exogenous emotional and physical sources [16,63,64,65]. Endogenous factors include genetic disposition, culture, personal attitudes, and perceived loss of control—when perceived control is affected by exogenous factors including the attitudes of close associates, and the effect of current and previous experiences on perceived control [26,64,65]. Such exogenous factors are open to manipulation, whether deliberate, through policy change, or otherwise, with positive or negative impacts [66].

### 3.1. Conversion of Stressors to Stress

The conversion of stressors to stress depends on coping capacity, stressor characteristics, personal disposition, learnt strategies, resource access, access to social support and context [23,62,67,68,69,70,71,72]. Inadequate or excessive adaptive responses can lead to endocrine, metabolic, autoimmune, and psychiatric disorders [16]. People in poor socioeconomic circumstance are more likely to suffer stressors and to have less opportunity and resources to cope with stressors [64]. Aboriginal people, and Indigenous peoples in general, are likely to suffer higher levels of stress than others in similar socioeconomic circumstance because of the additional range of stressors due to their Aboriginality. These include social, cultural and physical disenfranchisement [69,70,71,72] and limited access to resources [67].

### 3.2. Stress Leads to Two Interrelated Sets of Outcome

Stress includes two sets of outcomes. Firstly, when extended stress leads to disruption of the homeostasis system and to noncommunicable disease, loss of resistance to contagious disease and to cognitive disorders [18,63]. The second set of outcomes are risky behavioral responses, including lack of exercise, smoking, excess alcohol and poor food selection—although consideration of selection of food choice needs to take account of inadequate availability and the higher cost of good fresh food choices when living in remote areas. Remembering that risky behaviors are often the result of chronic stress, they are a secondary rather than a primary causative agent. Even when people are fully aware of the possible health impacts of risky choices, they may continue to engage in risky behaviors as a result of overwhelming stress [73].

As an example of the role of the psychosocial stressors through loss of personal control, the British Whitehall studies showed diabetes to be inversely related to the British Public Service hierarchy. The Whitehall I studies showed risky health choices explained less than 25% of diabetes incidence. The Whitehall II studies showed loss of control with decreasing public service status, explained more than 50% of diabetes incidence, with risky behavioral choices being, at least in part, a secondary response to primary psychosocial stressors [21]. Similar results have been observed for Australian and Canadian Aboriginal peoples [17,18,19,20,74,75]. It is important to recognize that the combined experience of Indigenous and non-Indigenous peoples is relevant when assessing the causative relationship between the primary causative stressors and health outcomes.

As many of the social connections held by Aboriginal people interconnect with country, dispossession from country can erode social connections and lead to negative health outcomes. Participation in traditional land management practices provides cultural and personal strength and control. An accumulating literature shows a positive correlation between participation in caring-for-country, control and health [31,32,33,34,35,36].

Fleming and Ledogar [76] and Ledogar and Fleming [77], for example, showed that for Australian and Canadian Indigenous peoples, cultural interaction enhances self-identity, confidence, behavioral norms and resilience. Cultural strength has been observed to provide the emotional strength for Aboriginal and Torres Strait Islander peoples to engage in western work and education [78,79,80].

Campbell [25] used an economic optimization model to explore whether the selection of poor health choices by Aboriginal people could be economically rational. In doing so, he reviewed an extensive literature showing those suffering high stress levels and uncertainty over future outcomes are less able to assert long-term self-control. This was shown to lead to a higher discount rate being placed on future outcomes, such as the possible benefits of self-investing in human capital, including healthy behavioral choices and education [81,82,83,84]. It was concluded that under chronically stressful circumstance, it can be economically rational to choose short-term risky behavioral choices that have a certain short term outcome, in preference to uncertain longer-term benefits, as through education. The solution to such behavioral responses is to alter the incentive structure through the removal of the psychosocial stressors [25].

## 4. Cost Savings through Caring-for-Country

### 4.1. Biopathological Assessment

Participation in caring-for-country associated with homeland residency in small family groups is shown to provide improved control and cultural and emotional strength [36]. The expectation for Aboriginal people to have the choice of remaining on traditional country has been criticized as unrealistic [60,85,86,87]. The following studies of Aboriginal communities in remote to very remote Northern Territory provides a counter argument to this view.

The change in mental and biophysical health status following the movement of Aboriginal people off the multi-tribal Papunja government settlement onto traditional homeland at Kungkayunti, or Brown’s Bore, provides an early example of the benefits of being on country [36]. There was an observed reduction in risky health behaviors. Three factors important to these changes were improved ego identity and improved self-esteem, opportunity to establish self-control, and, finally, the increased role of traditional doctors in addressing psychosomatic and psychic dysfunction [36].

Later studies include those by McDermot *et al.* [34] and Rowley *et al.* [35], in relation to the Algawarr and Anmatyerr peoples living on traditional homelands on what was the Utopia pastoral station and adjoining un-alienated country, in central Australia. The McDermott *et al.* [34] study involved Aboriginal residents in Hermannsburg and Utopia, while the follow-up study by Rowley *et al.* [35] was limited to the Utopia community. McDermot *et al.* [34] examined the association between high biological risk factors according to residence in the Utopia and Hermannsburg communities over seven years to 1995. Homeland residents were observed to have lower mortality, hospitalization, hypertension, diabetes and injury levels than settlement residents.

The 10 year follow-up by Rowley *et al.* [35], based on a cohort of McDermott *et al.*’s [34] Utopia population sample, supports McDermott *et al.*’s [34] results. Utopia residents were observed to have lower mortality rates than the Northern Territory Aboriginal population as a whole, even though their socioeconomic status was lower. This, it was suggested, is due to positive psychosocial responses attributed to increased personal control and connection to country and family. That the managers of the Utopia property were more accepting of traditional cultural connection to country is also likely to have been important [88].

The final example, in the West Arnhem Land Northern Territory top-end, involved traditional land owners and a multidisciplinary team of medical, ecological and social researchers [31,33,36]. Important to this study is the interrelationship between caring-for-country with an inverse association between caring-for-country and diabetes, hypertension, and renal disease [31,33]. Burgess *et al.* [31] noted homeland residents as being less likely to participate in risky health behaviors.

### 4.2. Economic Assessment

Two economic studies were carried out using data from the above studies. The first of these, by Campbell *et al.* [89], used Burgess *et al.*’s [33], West Arnhem Land data, to estimate primary health care cost savings for diabetes, renal disease and hypertension according to participation in caring-for-country. Cost data are from Zhao *et al.* [90]. The results showed potential annual savings of $268,000, for an eligible population of 1284 Aboriginal people aged between 15 and 55 years, with an estimated present value of savings over 25 years of $4.08 million. No allowance was made for the social benefit of a longer more productive and satisfactory life, savings in ongoing medical, transport and hospital costs, or for environmental benefits.

In a later study by Campbell [91], the West Arnhem Land results were compared with possible savings in central Australia on the basis of the incidence of diabetes and hypertension according to differences in body mass index (BMI), which differed according to township residence or homeland . The central Australian BMI results were respectively 25.7 (6.1), 23.5 (5.7) (standard error). This 9% difference indicates a lower health risk for homelands residents [34]. These results indicate the broader applicability of the Arnhem Land results. Based on an equivalent population size, the possible annual cost savings in central Australia were estimated to range between $160,443 (severity of disease level 1) and $268,137 (severity of disease level 2). Estimated annual cost savings for these conditions in Arnhem Land was $192,030. While a range of confounding factors can have influenced these results, failure to respond to these results could be a greater error [13].

### 4.3. Economic Characteristics

Recognition of the economic characteristics is important in optimizing the benefits of nonmedical primary-preventative interventions in comparing its cost effectiveness with alternatives, such as curative interventions. The benefits of caring-for-country include the Aboriginal community’s intended private goods benefits, and the jointly produced public goods (or bads) that provide national social benefits (disbenefits) (Table 2), These social benefits occur as unintended byproducts, or externalities; that is, without accompanying negative byproducts, or “bads”, these byproducts occur at no cost to society. Private good benefits consist of consumables, cultural connection and access to arts and crafts production. An often missed aspect of the indigenous art industry is that it provides a culturally intimate opportunity for people, who are otherwise unprepared for work, to earn income [92].

Private goods are rivalrous in consumption, with consumption by one person reducing the amount available to others. Public good benefits are non-rivalrous in consumption, which, in this instance, are socially beneficial. Some economists also require the additional condition that it is not possible to provide such goods through the market. As long as the social marginal benefit in the long run supplying the good exceeds the social marginal cost in the long run, an economic efficiency argument to supply the good through the market does not necessarily exist. Meeting such a requirement can result in economic inefficiencies. A distributional argument could, however, apply if higher income earners are the major beneficiaries.

Three sets of public good benefits are identifiable on this basis (Table 2). The first of these are the Aboriginal health benefits, as when these benefits go towards meeting the Council of Australian Government’s policy goal of closing the gap in Aboriginal social disadvantage [93]. The second set is the national health benefits, through the mitigation of airborne particulate matter and pathogens affecting East and West Coast populations. Environmental benefits make up the final set of public good benefits including biodiversity and the biosequestration of greenhouse gases.

Scoping economies and economic complementarities are important in affecting caring-for-country benefits. Scoping economies occur when the joint supply of two or more products is less than what they would be if they were supplied separately. Assuming no negative byproducts through caring-for-country, public good social benefits are provided at zero cost, and total marginal social benefit will exceed marginal private cost. Scoping economies are a result of joint or technical interdependence in production, or through economic interdependence, “… created by non-allocable inputs or linkages created by allocable fixed or quasi-fixed inputs” [94] (p. 10).

Complementarities occur when, firstly, the benefits of two or more components of a whole make-up for deficiencies in the other. This is the case when appropriate institutional structures create the incentives necessary for private investment in caring-for-country—as with private payment for biosequestration of greenhouse gases—or with the joint provision of preventative health and curative health in reducing the incidence and effect of chronic disease. Complementarities can be observed to exist when a reduction (increase) in the price of one good, or input, leads to an increase (decrease) in the demand for the other [95,96]. Supply side complementarities depend on the degree of non-substitutability between at least two inputs [97]. Regarding caring-for-country, cold weather use of fire is a complementary input to hunting and to land management, in addition to the scoping economies achieved through biosequestration, in aiding hunting and in maintaining biodiversity [34,98]. Indeed, the whole concept of “healthy country, healthy people”, is an example of complementary economies.

## 5. Optimizing Social Benefits through Caring-for-Country as a Preventative Intervention

### 5.1. Conditions for Optimality

The optimal allocation of resources across preventative and curative health is affected by government failure and/or market failure. Optimality exists when marginal cost intersects average cost from below and short-run and long-run functions intersect at the same point—thus meeting the necessary long-run and short-run marginal conditions. This requires the economic system to be in equilibrium. As economies are rarely, if ever, in equilibrium, this is neither likely, nor necessarily desirable [13]. It is appropriate, however, for policy objectives to be directed at, if not actually achieving, this objective.

### 5.2. Explaining Government and Market Failure

#### 5.2.1. Government Failure

The preference for curative health can be partly explained by a political response to individual preference for certainty in receiving treatment for specific conditions, rather than the personal uncertainty of population-based preventative intervention [13,14,99,100]. Implicit to this is that people may believe that the allocation of resources between preventative and curative health is rivalrous. If so, this response is akin to a minimax strategy, when people assume the worst in conditions of uncertainty and maximize according to what they know. Any rebalancing of health policies that engage with nonmedical primary-preventative health interventions requires a fully informed public. This is particularly relevant, if lack of information is important to people taking a minimax approach.

Health policy can be further distorted with suggested political competitiveness being an incentive for “bigger and better”, and “newer and more expensive” curative technologies [100]. The $20 billion Research Future Fund, proposed in the Australian Commonwealth Government’s 2014–2015 Annual Budget [101], and legislated in September 2015, being a possible example. Also relevant to possible distortion of government funding is lobbying by those with economic interests in curative health; e.g., pharmaceutical and medical manufacturers, hospital interests and general practitioners—especially given the gatekeeper role in primary health care of general practitioners [8].

An alternative or accompanying explanation is the existence of high discount rates by individuals or by government [102], which is likely to elicit a response in favor of acute health care in preference to preventative health. The focus on interest rates can be misleading. The short election cycle, by acting as a time constraint, can be a major cause affecting government policy choice, rather than what Lawless [102] attributes as being due to a high discount rate. A likely consideration affecting the focus on curative health is the training of those making and/or advising on policy decisions. That is, when those principally called on to provide policy advice come from a biological educational background.

An economically rational response, and one requiring careful explanation to the public, would be to allocate resources according to an economic criterion that is neutral in the maintenance of health. Allocating resources according to marginal cost-effective-need might go to meeting this. Any such analysis would need to take into account any nonhealth joint products, as observed with caring-for-country. As data on all aspects of such a program may not be readily available, all that is necessary is to show whether one approach is likely to be preferable to the other, or to narrow the range of unknown benefits and costs. This was the approach undertaken in the initial Hells Canyon analysis, which, as in this case, was to do with nonmarket based social values [103].

#### 5.2.2. Market Failure

When concerning market failure, the private provision of jointly provided private goods and public goods is likely to be sub-optimal. This can occur because of cultural differences, as when what Aboriginal people are seeking through caring-for-country does not match the expectancies of the wider community; e.g., protection of threatened species is seen as a foreign and unwarranted concept—or, alternatively, when people lack a broader view of recent changes, such as when they are unaware of the conditions that existed prior to feral animals [104]. Any such difference is likely to affect both the nature of and the level of private initiated investment in caring-for-country. Market failure can also occur when there is limited access to traditional country, or people are inhibited from participating in caring-for-country activities due to perceived or actual lack of mastery and control, [105]. Finally, market failure can occur when the private benefit of participation in caring-for-country is optimal, yet the combined marginal private and social public good benefits exceed the marginal cost of caring-for-country, such that the supply of these joint products is non-optimal. This is especially likely to occur when social public goods are provided as a byproduct at zero cost. As the public are the beneficiaries of these public goods, an economic argument can be made for public funding to enhance access to country, assertion of control, and extending caring-for-country.

The costs incurred in providing additional public funding by a single provider may be excessive. One approach is for each multijurisdictional beneficiary to fund incentives according to the relative benefits received. A multidisciplinary, multijurisdictional economic welfare framework can be used to assess the relative benefits using Black’s [106] cost effectiveness plane [107]. A negative aspect in a multijurisdictional approach is that costs are likely to increase as the number of jurisdictional partners increase; that is, transaction costs are likely to increase with increasing jurisdictional partners, in addition to possible loss of scale economies.

### 5.3. A Summation of Savings

As previously mentioned, an important factor affecting the cost effectiveness of primary-preventative health is the compression of morbidity with increasing age [40]; that is, since the 1950s, primary intervention has been observed to lead to an increase in modal length of life, a decrease in the standard deviation of age of death, and the delay of morbidity till close to death. Mur [45], while noting a general acceptance of the hypothesis, queried whether the global obesity epidemic could change this relationship. The Association of Faculties of Medicine of Canada [39] noted a positive relationship between life extending activities and improved health outcomes, so as to extend the “disability-free survival curve”. Important, as a result of compressed and decreased morbidity, is a likely decrease in misprescribed pharmaceutical and clinical interventions resulting in a further decrease in morbidity and mortality [108].

One issue in financing a rebalancing of resources to accommodate primary preventative health is the time lag that will occur before any reduction in demand for curative health occurs. This means the initial period of time during which the effect of primary-preventative health works its way through the population will require additional funding. Such changes will lead to additional improvements in human capital and social wellbeing, and savings in pharmaceutical and clinical costs and related treatment, including hospitalization.

## 6. Examples of External “Incentive” Payments

In addition to individual self-interest, there has been a history of ongoing public and private funding for Aboriginal involvement in caring-for-country. These include private, state, territory and commonwealth payments and transfers. In addition to other programs, the Commonwealth Government’s Indigenous Land Corporation provides funding for the purchase of country for communities who are unable to gain rights over traditional country through the Native Title Act 1993. This is provided for purpose of protecting cultural and environmental values, and enhancing Aboriginal and Torres Strait Islander socioeconomic development. The Department of the Environment carries out joint programs providing funding for the involvement of traditional owners in traditional land management programs on country. These range from specific programs, such as a “two-way” botanical survey involving Indigenous and western ways of identifying and classifying Arnhem Land native species [109] and funding for Indigenous Protected Areas under the National Reserve System. The budget for the current five year Indigenous Protected Areas program, starting in 2013–2014 was set at $78.3 million. Indigenous protected areas make up approximately 40% of the total National Protected Area Program of 137.5 million hectares. Such programs also provide culturally acceptable employment in locations in which employment opportunities are limited—with consequent, and unaccounted for, social and health benefits.

Traditional cool weather burning has potential for commercial development of carbon credits. An early Australian program started in 2006 with the establishment of a 17-year agreement in West Arnhem Land involving traditional land owners, the Northern Territory Government and Conoco-Phillips/Santos. Intended to offset greenhouse gases generated through the establishment and running of a liquefied natural gas plant in Darwin, it involves an annual $1 million fee paid by Conoco-Phillips/Santos to fund joint traditional and western land management practices. Intended benefits are the annual biosequestration of greenhouse gases equivalent to 100,000 tonnes of CO_2_, the protection of fire-threatened plant species and local employment of traditional owners [110].

The Fish River property of 178,000 hectares, owned by the Indigenous Land Corporation, north-western Northern Territory provides another example. This involved the sale of 25,884 Australian Carbon Credit Units at $20 per tonne of CO_2_ equivalent to Caltex Australia Oil Co [111]. This and subsequent carbon credit agreements with traditional land holders, were carried out under the Commonwealth Government’s Carbon Credits (Carbon Farming Initiative) Act 2011. Greenhouse gas abatement levels of these programs are expected to range between 25% and 48%, with efficacy of cool weather burning varying according to location and seasonal variation [112]. While these programs resulted in multiple benefits, including health, environmental benefit was the primary policy objective.

## 7. Conclusions

The starting point for this paper is the imbalance between non-medical primary-preventative health and curative health, and extending the applicability of non-medical primary-preventative health to the global noncommunicable disease pandemic. Two case studies, one in tropical northern Australia and the second in the central Australian desert, were used to exemplify the broad social benefits of a nonmedical primary-preventative health approach to the mitigation of noncommunicable disease. 

The two case studies demonstrated the possibility of substantial savings in primary health care, the implied medical savings in foregone hospital treatment and patient air evacuation to and from hospital care. These savings occurred in addition to the private, family and Aboriginal community benefits of healthy members. A range of private and public good social benefits are shown to be achieved through caring-for-country, with the public good social benefits provided at zero cost as a byproduct of caring-for-country.

A theoretical public economics framework is used to examine the economic structure of the case studies. This allowed identification of the private good and social public good benefits, the respective roles played by the private and public sectors, and how this knowledge could be applied in achieving optimal outcomes. This approach allows recognition of multiple benefits (disbenefits) and the multidisciplinary, multijurisdictional interests of these benefits. These aspects are important to the cost effectiveness of non-medical primary-preventative health. Relevant to this are the possibilities of scoping economies and the presence of complementary economies between nonmedical primary-preventative health and curative health. Awareness of the probable existence of such economies is important in designing, applying and estimating the relative benefits of a non-medical primary-preventative health intervention, and in optimizing the social benefits within the national budgetary constraint.

Applying nonmedical primary preventative health will differ between population groups according to location and context, including the culture and history of the people involved. In addition to likely savings in health costs, a rebalancing to primary preventative health is likely to result in a more productive population due to extended life and morbidity compression; that is, people can be expected to live and work longer, and the demand for curative services to be reduced and discounted into the future. Such outcomes are important in developed and developing economies alike, especially if the full national social benefits of human capital and extension of the tax base are realized—an especially important point of consideration among aging national populations.

Consideration of the health and broader benefits of Aboriginal people in caring-for-country demonstrate the possible advantages of primary preventative health in general. In doing so, it also shows that support of programs based on caring-for-country is not something “special” or covert welfare for Aboriginal people. Instead, it exemplifies the application of primary preventative health according to the context of the population involved. A possible urban example might relate to the relative benefits of rail *versus* road infrastructure when there are direct health issues of injury and pollution, and mass movement of people and goods under conditions of limited space, average time spent travelling and distributional impacts according to cost and time spent—especially given that the less wealthy are more likely to be located in the outer suburbs, and loss of time through extended travel are added to individual social disruption.

In conclusion, the material presented here provides a strong economic argument in favor of consideration of traditional caring-for-country practices, especially among Aboriginal people in remote to very remote Australia, as well as to Indigenous people in general. At a higher level of abstraction, these results have been used to show how nonmedical primary-preventative health interventions can be applied as a cost effective means to the prevention and mitigation of the global chronic disease pandemic.

Achieving a systematic coordinated balance between curative and nonmedical primary-preventative health will require research that accounts for the particular complementarities between preventative and curative health. As suggested, public support for the introduction of nonmedical primary-preventative health requires the public to be fully informed if the political support for the application of nonmedical primary-preventative health might occur.

## Figures and Tables

**Table 1:** Age standardized relative indicators of Indigenous noncommunicable disease burden [37].

Year/s	Condition	Rate Relative to Non-Indigenous
2012	Death rate	1.6 times that of non-Indigenous
2010–2012	Life expectancy	10–11 years less than non-Indigenous
2012	Diabetes death rate	7 times that of non-Indigenous
2009–2012	End stage renal disease	6.2 times that of non-Indigenous
2012–2013	Hospitalization for injury	~Twice that of non-Indigenous
2012–2013	Respiratory condition	1.2 times that of non-Indigenous
2012–2013	High to very high levels of Psychological stress	2.7 times that of non-Indigenous

**Table 2:** Joint products originating from Aboriginal traditional involvement in caring-for-country in remote to very remote Australia [91].

Private Good
Aboriginal community benefits:
- Traditional foods, medicines and materials
- Meeting community based cultural responsibilities
- Health, including compressed morbidity & extended life
Public Good
National health (environmental) benefits:
- Mitigation of dust storms through cold weather burning
- Mitigation of excess smoke and particulate matter
Public Good
Environmental benefits:
- Biodiversity
- Biosequestration of greenhouse gases
- Soil stabilization
- Mitigation of dust storms
Public Good
Aboriginal health benefits:
- Compressed morbidity & extended life; e.g., direct: traditional foods, medicines & exercise
- Psychosocial determinants; e.g., meeting cultural responsibilities & elements of wellbeing
